# Evaluation of the Use of Cell Lines in Studies of Selenium-Dependent Glutathione Peroxidase 2 (GPX2) Involvement in Colorectal Cancer

**DOI:** 10.3390/diseases12090207

**Published:** 2024-09-10

**Authors:** R. Steven Esworthy

**Affiliations:** Department of Cancer Genetics and Epigenetics, Beckman Research Institute, City of Hope National Medical Center, Duarte, CA 91010, USA; sesworthy@coh.org

**Keywords:** selenium-dependent glutathione peroxidase, peroxiredoxins, NADPH oxidase 1, colorectal cancer, cell lines, public databases

## Abstract

Hydroperoxides (ROOHs) are known as damaging agents capable of mediating mutation, while a role as signaling agents through oxidation of protein sulfhydryls that can alter cancer-related pathways has gained traction. Glutathione peroxidase 2 (GPX2) is an antioxidant enzyme that reduces ROOHs at the expense of glutathione (GSH). GPX2 is noted for a tendency of large increases or decreases in expression levels during tumorigenesis that leads to investigators focusing on its role in cancer. However, GPX2 is only one component of multiple enzyme families that metabolize ROOH, and GPX2 levels are often very low in the context of these other ROOH-reducing activities. Colorectal cancer (CRC) was selected as a case study for examining GPX2 function, as colorectal tissues and cancers are sites where *GPX2* is highly expressed. A case can be made for a significant impact of changes in expression levels. There is also a link between GPX2 and NADPH oxidase 1 (NOX1) from earlier studies that is seldom addressed and is discussed, presenting data on a unique association in colon and CRC. Tumor-derived cell lines are quite commonly used for pre-clinical studies involving the role of GPX2 in CRC. Generally, selection for this type of work is limited to identifying cell lines based on high and low *GPX2* expression with the standard research scheme of overexpression in low-expressing lines and suppression in high-expressing lines to identify impacted pathways. This overlooks CRC subtypes among cell lines involving a wide range of gene expression profiles and a variety of driver mutation differences, along with a large difference in *GPX2* expression levels. A trend for low and high *GPX2* expressing cell lines to segregate into different CRC subclasses, indicated in this report, suggests that choices based solely on *GPX2* levels may provide misleading and conflicting results by disregarding other properties of cell lines and failing to factor in differences in potential protein targets of ROOHs. CRC and cell line classification schemes are presented here that were intended to assist workers in performing pre-clinical studies but are largely unnoted in studies on GPX2 and CRC. Studies are often initiated on the premise that the transition from normal to CRC is associated with upregulation of *GPX2*. This is probably correct. However, the source normal cells for CRC could be almost any colon cell type, some with very high *GPX2* levels. These factors are addressed in this study.

## 1. Introduction

Selenium-dependent glutathione peroxidase 2 (GPX2) is a member of the GPX family of antioxidant enzymes that reduce peroxides such as H_2_O_2_ and fatty acid hydroperoxides (ROOHs) at the expense of the tripeptide glutathione (GSH) [1]. When GPX2 is expressed, it is generally in the company of its sister isoenzyme, GPX1, and the peroxiredoxin (PRDX) family of ROOH reducing activities that use thioredoxin as a reducing agent. Since the initial characterization of *GPX2* gene expression in the gastrointestinal (GI) tract and other human and rodent tissues, many studies have examined the roles of GPX2 in carcinogenesis in epithelial cells [1,2]. Too often, the full context of antioxidant enzyme families is overlooked, leading to claims that GPX2 is having strong effects in cases where its expression is low by comparison [1,2]. There may be a basic misconception that the ability to detect the mRNA by RT-PCR, the protein by Western blotting, or by immunohistochemistry (IHC) represents significant expression levels by not examining GPX1 or PRDXs [1,2]. In several studies using RT-PCR, *GPX2* levels seemed to be at the lower limits of detection (sometimes by comparison to cell lines with established high *GPX2* expression levels), and yet the protein was still detectable on Western blots, encouraging the view that GPX2 could be exerting a significant impact in this lower range [1,2]. In one instance, GPX2 was shown to be detected in glioblastoma multiform (GBM) by IHC from The Human Protein Atlas (THPA), a cancer that barely expresses *GPX2* [1]. The Human Protein Atlas warns that GPX2 antibodies used in that analysis appear to exhibit off-target binding (https://www.proteinatlas.org/, as of 30 May 2024) and declares that, generally, the protein was not detected in GBM, while in the detailed comments suggesting that Purkinje cells and not glia show some GPX2 staining in normal samples.

**Figure 1:**
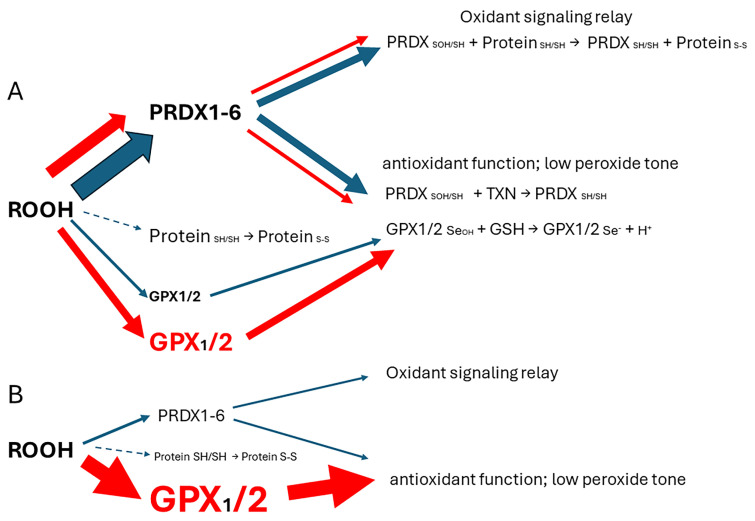
(**A**) GPX1 and GPX2 are in competition for ROOHs with PRDX. The greater abundance of PRDXs in most cell types and the higher rate constants of PRDX1 and PRDX2 (2–5-fold) would divert a major portion of ROOHs into a signaling relay in combination with a general antioxidant function (Blue arrows, default pathways flux), largely preventing direct oxidation of proteins (dashed arrow) [3]. The reduction of oxidized PRDXs may be slow (interaction with protein targets and bottleneck at thioredoxin reductase; thioredoxin, TXN; glutathione, GSH), so that GPX1 and GPX2 take up the slack while PRDXs regenerate back to the reduced form, maintaining the required low peroxide tone [4]. When GPX2 and/or GPX1 attain higher relative levels (RED, and RED arrows; pathways flux after elevation of GPX1/2), they would siphon ROOHs from the PRDX oxidant relay and alter the impact of ROOH signaling. (**B**) In the GI tract, where a few cell types have very high levels of GPX2, there may be a dependence on GPX1/2 over and above that of repressed PRDXs to prevent direct oxidation of cell components by ROOHs, demonstrable when *GPX1* and *GPX2* are knocked out in mice, leading to excess apoptosis and anoikis, the latter responsible for inflammation of the ileum and colon.

GPX1 with a nearly identical hydroperoxide substrate range was not included in most of these studies. This is unfortunate because *GPX2* expression is often minute compared to *GPX1* (GBM) or at best comparable, and the sum of both is often small compared to the ubiquitous peroxiredoxin antioxidant collective (*PRDX1-6*) [1]. Thus, GPX2 seems to be exerting effects, which are not truly explained despite the apparent variation in cellular oxidant levels after manipulation of *GPX2* levels [1]. Colon/rectum and colorectal cancer (CRC) are cases where GPX2 levels are high enough to have an impact on ROOH levels. In the overall scheme of ROOH antioxidants, GPX1 and GPX2 seem to act solely as reducing agents, maintaining a low peroxide tone. In this context, low peroxide tone means preventing direct oxidation by ROOHs and damage to DNA, cell membranes, and proteins. PRDXs are postulated to have two general functions. In competition with GPXs, PRDXs would be acting as general antioxidants. Second, PRDXs may act as oxidant relays, eliminating ROOH species as potential damaging agents and safely registering and conveying the oxidant signal as PRDX-SOH to target proteins (Figure 1) [3,4]. PRDX1 and 2 would tend to dominate ROOH metabolism by virtue of high levels of expression and initial ROOH rate constants greater than GPX1 and GPX2. The mechanism of action of GPX1 and GPX2 at high levels of expression in this model is diversion of ROOH away from the PRDX relay, thereby nonspecifically lowering the impact of ROOHs on signaling. This is a real possibility in CRC, esophageal, and stomach cancers, as shown in this report. Possible involvement of *GPX2* in other cancers was evaluated in ref. [1]. The downstream effects and specificity would be dependent on the general gene expression profile of the cell types. In CRC, as many as six general expression profiles are noted. Thus, different downstream effects could arise from the impact of GPX2 based on widely different general gene expression profiles and not a direct, specific effect of GPX2. In this investigation, cell lines are sorted with the finding that cell lines with low and high *GPX2* levels end up in different profile categories. While this is of interest, the very low expression in some cell lines is at odds with the expression levels of the corresponding CRC types, calling into question their value in studies. A common trope in studies on GPX2 roles across many cancer types is overexpression in low-expressing cell lines and reciprocal knockdown in high-expressing cell lines [1,5]. Thus, different downstream effects could arise from the impact of GPX2 in each category based on widely different general gene expression profiles and not a direct, specific effect of GPX2. The goal is to provide some guidance in selecting CRC-derived cell lines to study colorectal cancers in the context of *GPX2* expression, specifically selection of certain cell lines to accomplish study of CRC types that tend to relapse and metastasize, avoiding use of cell lines with exceedingly low *GPX2* levels. The histological and functional links between superoxide-generating NADPH oxidase 1 (NOX1) and GPX2 have been demonstrated in colon/rectum, CRC, and cell lines and are discussed in the context of cell line choices.

## 2. Materials and Methods

The primary tool used in this study was internet access to public databases, the Cancer Dependency Map consortium (DepMap, https://depmap.org/portal/; 30 January 2024), The Human Protein Atlas/The Cancer Genome Atlas (THPA/TCGA, https://www.proteinatlas.org/; https://www.cancer.gov/ccg/research/genome-sequencing/tcga; 30 January 2024), Gene Expression Profile Interactive Analysis (GEPIA2, http://gepia.cancer-pku.cn/; 30 January 2024), Tumor IMmune Estimation Resource (TIMER2.0, (http://timer.cistrome.org/; 30 January 2024), and PubMed (https://pubmed.ncbi.nlm.nih.gov/; 30 January 2024 to 2 June 2024). Analysis spanned the time frame of 30 January 2024 to 2 June 2024). The use of data from these sites was previously explored and validated against results from experimental studies, with some discrepancies noted [1]. The extrapolation of TPM to protein levels was examined and found to be reasonably consistent [1]. The analysis relied on basic tools accessible to most investigators. Excel MSO version 2403 was used to collate and process datasets and generate tables and some graphs, GraphPad PRISM 9.3 was used to generate graphs and perform statistical analyses, and Microsoft Power Point was used to create figures. The primary analyses involved sorting CRC-derived cell lines, focusing on *GPX2* expression levels, into CRC categories based first on marker analyses in Medico et al. that provided the biggest set of cell lines (150 with 65 of 77 from DepMap found in common; 30 January 2024) [6]. While starting with the methods of Sadanandam et al., they sorted additional cell lines with some new markers and weighting [7]. Then, they sorted the 150 cell lines based on the analyses of Marisa et al., Budinska et al., De Sousa E Melo et al., and Roepman et al. [8,9,10,11]. CRC subtype analysis and corresponding cell lines were taken directly from Schlicker et al., Sadanandam et al., Berg et al., and Hu et al. (mucinous adenocarcinoma markers) [7,12,13,14]. *GPX1*, *GPX2*, *NOX1*, *PRDX,* and *CAT* expression data for cell lines was from DepMap. Because only one value is provided for each cell line, THPA’s somewhat smaller list was consulted to get some sense of the inherent error (56 cell lines in common) for *GPX2* and *NOX1*. In the text, cell line *GPX2* expression levels are presented as the average of DepMap and THPA when both are available. In the figures, only DepMap values are presented, except to correlate with THPA. Marker analysis was performed using a short-hand list from the iCMS system and the combined expression data (average, log2 TPM+1 for markers) [15]. In addition, for HT29, markers associated with differentiation (*MUC2* and *LYZ*), TGFB expression (*CCN1*, *ITGAV*, and *TGFB*), and EMT (*FN1*, *CDH2*, and *VIM*) were examined [16,17,18]. Statistical analysis was performed for *GPX2* expression levels among CRC subtype groupings, Mann–Whitney, log2 transformed data directly from DepMap, and PRISM 9.3.

## 3. Results and Discussion

### 3.1. Correlation of GPX2 Levels in Tumors and Cell Lines

**Figure 2:**
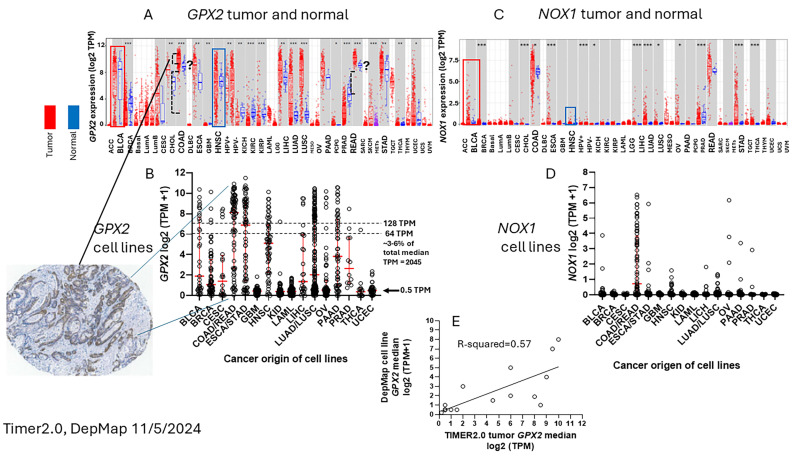
Comparison of normal tissue and tumor sample *GPX2* (Panel (**A**)) and *NOX1* expression levels (Panel (**C**)) (log2 TPM) with levels in cancer-derived cell lines (Panels (**B**,**D**); log2 TPM+1) (TIMER2.0 and DepMap-11/5/24). *, **, *** in panels (**A**,**C**) denote significant differences between the expression levels of the respective normal and tumor samples as *p*-values, *, 0.05; **, 0.01; ***, 0.001. Each circle in panels (**B**,**D**) is a separate cell line; in panel (**E**), each circle represents median cancer sample and derived cell line values. The CRC tumor sample GPX2 IHC insert (THPA) is estimated to have *GPX2* expression levels falling inside the upper bracket (COAD tumors). The lower bracket denotes rare samples with very low expression. Low-expressing READ tumors are also bracketed. ? denotes high *GPX2* expression from a few normal cells in the colon. The dashed lines across the cell line *GPX2* expression panel denotes 2 thresholds for significant levels, relative to total antioxidant gene expression—*GPX1*, *GPX2*, *PRDXs 1–6*, and catalase—in TPM (2045 total TPM). The median *GPX2* expression level for all cell lines is 0.5 TPM. BLCA and HNSC tumor samples have broad ranges of *GPX2* expression (red and blue rectangles) along with LIHC, suggesting nothing anomalous with the isolation of both high and low *GPX2*-expressing cell lines, unlike COAD/READ. (Panel (**E**)) shows the correlation between the median tumor levels and the median cell line levels for each tumor type. The TCGA tumor abbreviation convention is used (https://gdc.cancer.gov/resources-tcga-users/tcga-code-tables/tcga-study-abbreviations accessed 30 May 2024).

**Figure 3:**
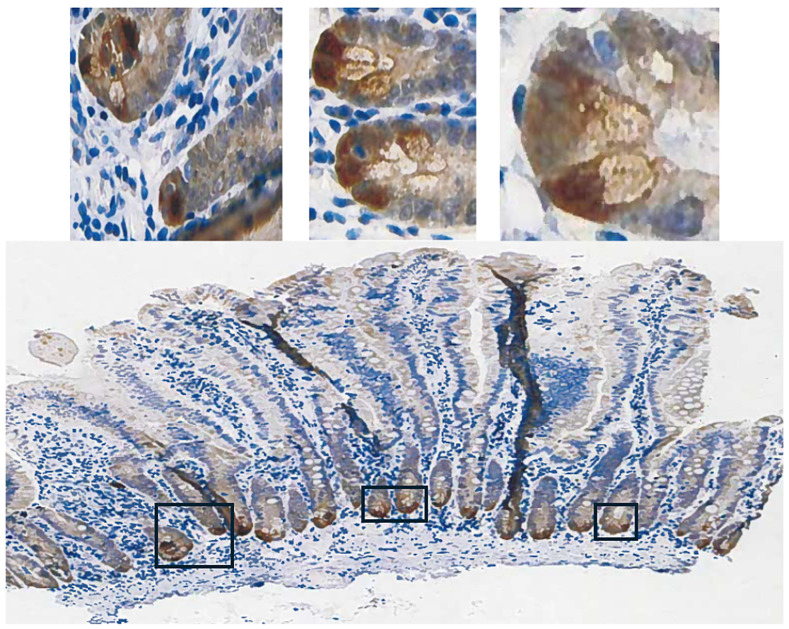
The few cells with high GPX2 protein levels in the small intestine are at the crypt base (lower panel) and appear to be Paneth cells (upper panels; black rectangles denote the boundaries of the upper panels from the lower panel). This localized high GPX2 expression may supersede PRDXs for overall antioxidant protection (Figure 1B). Some low-level expression may extend to the top of the crypt to include other cell types (THPA: NOS (M-00100) Patient id: 2411; 5 May 2024).

To ascribe the importance of antioxidant enzymes that reduce ROOHs, it is necessary to demonstrate that an enzyme is present in a significant quantity (Figure 1) [1,2]. The initial characterization of *GPX2* to be highly expressed in the gastrointestinal (GI) tract and undetectable in many other tissues by Northern blotting is still valid (Figure 2A) [1,2,19]. Based on results compiled by the TIMER2.0 database, in general agreement with TCGA (ref. [1]), *GPX2* is highly expressed at the tissue level in the mid-lower GI (corresponding cancer, TCGA abbr.; colon/rectum, COAD/READ; 457 COAD samples vs. 41 normal and 166 READ samples vs. 10 normal in Figure 2A; 30/5/2024), bladder (BLCA; 408 vs. 19 normal), esophagus (ESCA; 189 vs. 11 normal), head and neck tissues (HNSC; 520 vs. 44 normal), liver (LIHC; 371 vs. 50 normal), stomach (STAD; 415 vs. 35 normal), in cancerous pancreas (PAAD; 178 vs. 4 normal; median for normal is several logs higher than other database values; ref. [1]) and lung squamous cell carcinoma (LUSC; 501 vs. 51 normal), although at a lower level in lung adenocarcinoma (LUAD; 515 vs. 59 normal) (Figure 2A) [1]. High expression in normal tissues is often confined to a few cell types. The small intestine is a prime example of the limited zone of expression within a high-expressing tissue, apparently just the easily recognizable Paneth cells (Figure 3; THPA). This creates one of the problems in analyzing *GPX2* using tissue-level metrics from databases, as will be elaborated, and is not unique to the GI tract (Figure 2 and Figure 3) [1].

**Figure 4:**
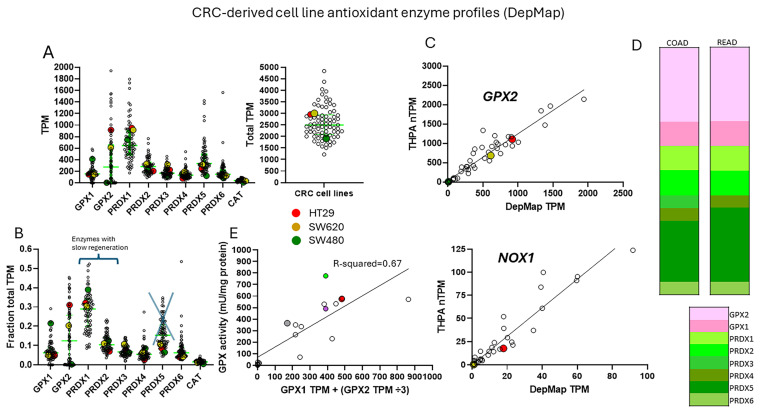
Antioxidant gene expression levels in CRC-derived cell lines (Panels (**A**,**B**); DepMap) with total levels (Panel (**A**)). Error bars are median levels and quartiles. The Xed out PRDX5 set indicates the low ROOH rate constant that basically factors PRDX5 from consideration in apprising impact (panel (**B**)). Slow reduction of oxidized PRDXs 1 and 2 may bring the enzymes back down into the overall ROOH rate of metabolism with GPX1 and GPX2, although their initial ROOH rate constants are greater than GPX1 and GPX2; see text. Panel (**C**) is an indication of the relative error in DepMap TPM values for *GPX2* and *NOX1* based on comparison with THPA nTPM as a correlation. The colorectal tumor panel shows the relative fraction of GPXs and PRDXs to total antioxidant mRNA expression in COAD and READ (Panel (**D**)). The oversized dots in the cell line (Panels (**A**–**C**)) represent 3 cell lines discussed in the main text: HT29 (red), SW480 (dark green), and SW620 (yellow). (Panel (**E**)) is a comparison of GPX1/2 activity levels in cell lines determined by the author in prior studies [1,2] with DepMap TPM using the following formula: GPX1 TPM + (GPX2 TPM ÷ 3) [1], including CRC-derived cell lines, HT29 and CACO2, red and gray dots. The intestinal cancer cell line, HUTU80, also assayed by the author, is at 1.3 TPM and 27 mU/mg. The green and purple dots are two activity measurements of OVCAR8 (NCI/ADR-RES) separated by four years.

Cell line *GPX2* gene expression levels were compiled based on tissue and cancer of origin (DepMap; 30 May 2024). As shown in Figure 2B, cell lines derived from COAD/READ as well as from ESCA/STAD have a high median level of *GPX2* gene expression, while many cell lines from HNSC and PAAD also have somewhat elevated *GPX2* expression, based on median levels. There is some correlation between the respective median tumor levels and median cell line levels (Figure 2E). The low *GPX2* levels in cell lines derived from the high-expressing cancer sources, COAD/READ and ESCA/STAD, are somewhat unique among the antioxidant enzyme genes *GPX1* and *PRDX1-6*. A few cell lines have lower than expected *GPX1* and *PRDX* levels, but nothing like *GPX2* (*GPX2*, 59/128 low expressing lines (≤64 TPM; ref. [1]); *GPX1*, 13/128; *PRDX1*, 0/128; *PRDX2*, 6/128; *PRDX3*, 3/128; *PRDX4*, 6/128; *PRDX5*, 0/128; *PRDX6*, 2/128; Figure 4) [1]. The low expression of *GPX2* in cell lines could be anticipated for BLCA, HNSC, LICH, and PAAD from the variation observed in tumors and is not explained by variation among COAD/READ and ESCA/STAD samples at the tissue level (Figure 2A).

For a variety of reasons, the relative TPM levels among tissues and cell lines presented represent only a rough guide to thinking about where GPX2 might have a significant role (Figure 2 and Figure 4). DepMap TPM results have some predictive value for GPX activity in cell lines (Figure 4E; selenium-supplemented culture media for cell line activity) [1]. GPX activity at and above 190 mU/mg is in the range of normal tissue and cancer sample levels [1]. One word of caution is made with regards to the *GPX* TPM data of DepMap and THPA and for study in almost all current papers [1]. The two database projects and almost all current published studies did not include supplementation of the culture media with selenium (10% FBS/FCS; fetal bovine/calf serum; some rare exceptions in DepMap in combination with low serum for culture) [20]. As a rule, this is suboptimal for GPX1 and GPX2 protein and activity levels in cell lines [1,2,21]. Different batches of serum can have widely different selenium levels, and this will have an impact on the protein and activity levels of both GPX1 and GPX2 and possibly on the mRNA levels of *GPX1* [21,22,23]. *GPX1* mRNA levels may be underrepresented in the databases and in studies [1]. Selenium supplementation could conceivably shift up to 8 of the 13 low-expressing *GPX1*-level GI tract-derived cell lines into the high expression range (2-fold increase; see HepG2 in ref. [22]). In studies, the protein and GPX activities will often be up to one-half to one-fourth the optimal levels and can be even less; the lower activity is a feature of cell lines expressing only *GPX1* [22,23]. This could impact the reproducibility of findings and downplay the role of GPX1 in favor of GPX2 [1].

### 3.2. The Choice of CRC-Derived Cell Lines for a Case Study: GPX2 and NOX1

It was reported that the superoxide-generating NADPH oxidase, NOX1, is a major source of oxidants that produce ileocolitis in mice deficient in both *GPX1* and *GPX2* gene expression [24,25]. While mice deficient in both *GPX1* and *GPX2* (i.e., GPX1/2-DKO; double knockout) have spontaneous ileocolitis (Figure 1B; supporting a lesser role for PRDXs in general antioxidant function), after knocking out *NOX1*, the triple KO mice no longer have gut pathology. Eliminating the H_2_O_2_-generating enzyme, DUOX2 (via a DUOXA-KO), relieved crypt/gland base anoikis and inflammation while having no impact on excess crypt/gland base apoptosis observed in the DKO mice. *NOX1* mRNA is uniquely highly expressed in normal and cancerous colorectal tissues (Figure 2C). The same pattern of *NOX1* expression is observed in cell lines derived from colorectal cancerous tissues (Figure 2D). It is likely that the indicated colocalization of *GPX2* and *NOX1* in the crypt/gland epithelium (although not necessarily the same cells) of the intestine and colon allows them to keep balance in the redox status [26,27,28].

Some cells in the colon epithelium have naturally high expression of *GPX2,* and this seems to carry over into the cancers [29,30,31]. The estimated levels of GPX2 at the mRNA and protein levels in CRC show that they likely exceed that of GPX1 and rival that of PRDX1 and PRDX2, meaning GPX2 could have a major impact on ROOH levels (Figure 4D) [1]. This and the apparent link to *NOX1* led to choosing CRC-derived lines as the study case for trying to understand what to consider in selecting lines for cancer studies. The complexity of issues in CRC and the peculiarities of cell line properties and culture methods make this a difficult case study. While this analysis may not completely succeed, the details of the process may be instructive.

### 3.3. Remaining Uncertainties in GPX2 Expression in Normal Colon/Rectum

**Figure 5:**
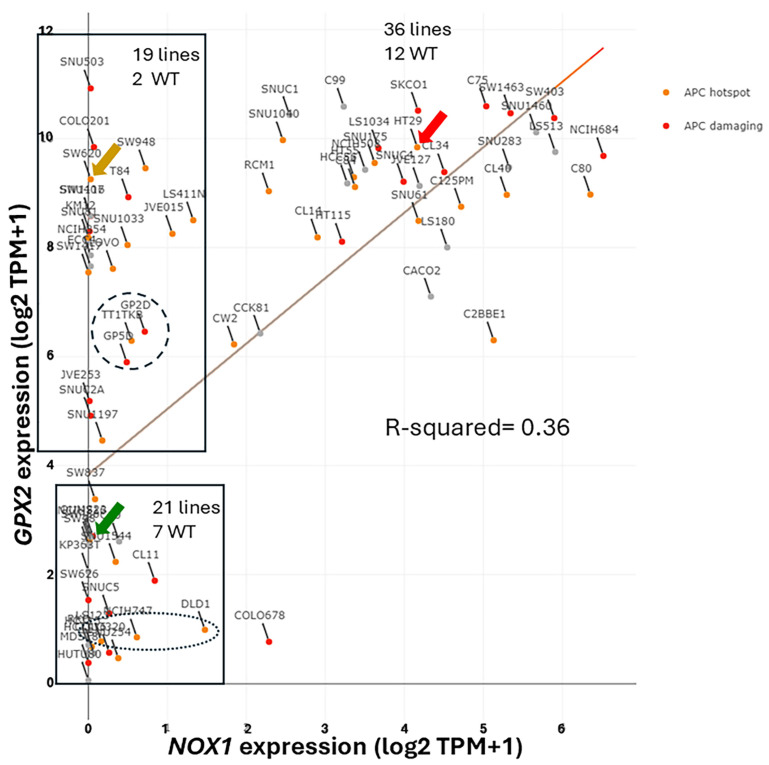
Correlation of *GPX2* and *NOX1* expression in the set of 77 CRC-derived cell lines in DepMap (30/5/2024), denoted by *APC* gene mutation status. Cell lines HT29, SW480, and SW620 are indicated with arrows. The dashed circle encloses paired cell lines, GP2D and GP5D (same primary), and the dashed oval the suspected DLD1 and HCT15 pair. WT-wildtype APC gene.

The reasons for lack of a clear answer to the choice of cell lines include no direct link between the cell types that naturally express *GPX2* at high levels and its expression in tumors. Despite decades of characterization, the exact cell expression profile in the colon is unclear [29]. With studies using single-cell profiling of normal colon and CRC available, it should be a simple matter to establish the identity of the normal high *GPX2* expressing cells and possibly infer *GPX2* expression levels for sources of CRC cells; this is based on data from polyps representing an early point in the malignancy continuum [15,32]. Variable co-expression profiles between *GPX2* and *NOX1* might aid in this process (Figure 2 and Figure 5).

The possible candidates for high *GPX2* expression are Paneth cells (Figure 3; mice develop tumors in the small intestine) or the colon equivalent, deep secretory cells (so far, identified in mice and not documented for *GPX2* expression) [33]. Refined localization of GPX2-expressing cells in the human colon by IHC shows that they represent only a few cells at the base of each gland, paralleling the small intestine (Figure 3) [29]. A single-cell analysis did confirm high *GPX2* expression in Paneth cells, while being somewhat unclear about other cell types, particularly in colon [34]. Paneth cells are not known to give rise to tumors, although they exhibit plasticity during loss of LGR5+ crypt cells that allows them to gain stem cell properties and repopulate crypts [35,36,37,38]. Whether they retained *GPX2* expression in this process was not determined. It is this type of plasticity exhibited across many cell types in the colon during carcinogenesis that lends itself to uncertainty over whether *GPX2* is being upregulated and to what extent [36]. Upregulation would be the default when any cell types outside of the lower crypt/glands are the source of the tumors (Figure 3) [39]. There is some evidence that upregulation of *GPX2* mRNA levels on the order of 4–5-fold in Stem-like cells occurs during the progress from early lesions to CRC [32]. Similarly, looking at whatever cells are expressing GPX2 protein at high levels in normal colon suggests possible elevation up to 5-fold in protein levels in CRC cells (statistically significant) [29]. However, that determination did not establish any link between the normal cells and the cell types that gave rise to the tumors. If the candidate cells are not the naturally high *GPX2*-expressing cells, the magnitude of elevation could be tens-hundred-fold, providing a strong rationale for GPX2 impacting tumorigenesis by modulation of ROOH levels. There is ample evidence that an alteration of *GPX2* levels from essentially no expression to levels characteristic of most CRC samples (~27% of total antioxidant enzyme TPM; GPXs, PRDXs, and catalase) would impact tumor pathways via known redox-sensitive proteins, such as PTEN (Figure 1A and Figure 4D) [1,40,41].

### 3.4. The Problems with Databases Combined with the Issues of Normal Cell Expression

**Figure 6:**
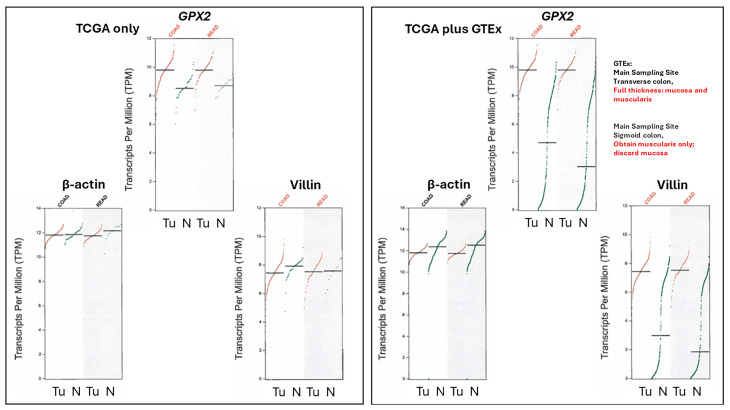
Discrepancies in the *GPX2* expression levels of normal samples in GEPIA based on database sources (TCGA alone or including GTEx). The red text indicates the 2 discrepant sample processing protocols mentioned in GTEx that might contribute to the outcomes. Other genes, such as *VIL* (villin), are apparently impacted by the alternative GTEx normal tissue sampling protocols, compared to β-actin.

Note, the upregulation of *GPX2* expression is not being based on data from sites like TCGA/THPA, TIMER2.0, and GEPIA2. GEPIA2 data present real problems with exceptionally low *GPX2* expression found in many normal samples (Figure 6). This extends to several other genes whose expression is confined to the mucosa as opposed to those expressed in the mucosa and mucularis, like villin vs. β-actin (Figure 6).

TCGA/THPA, TIMER2.0, and GEPIA2 tumor databases are consistent in showing CRC tumors as having high *GPX2* expression, with only a few exceptions (28/597 CRC samples TPM < 256, THPA; TIMER2.0 has ~18/623; Figure 2A; GEPIA2 ~12/397; Figure 6). The issue with normal samples in GEPIA2 might stem from the divergent protocols for processing between the two sampling sites mentioned in GTEx (https://gtexportal.org/home/ 30 January 2024), the source of much of the data. For transverse colon, full-thickness samples were analyzed, while for sigmoid colon, only the mucularis was sampled. *GPX2* is not expressed outside of the epithelium of the mucosa, except for scattered cancer-associated fibroblasts (CAFs) (Figure 3) [29,30,31,42]. It is not clear that this is a complete explanation for the discrepancy between the GEPIA2 data sets and TCGA/THPA and TIMER2.0. The rectum set does not mention similarly divergent processing protocols, and the sample numbers for the alternatively processed colon samples do not add up to the total number of samples listed. TCGA/THPA and TIMER2.0 show a much lesser range of increase in *GPX2* expression between normal and CRC, on the order of 2–20-fold (see matched sets, ref. [43]), with a few matched samples showing a decrease in CRC or no alteration [43].

**Figure 7:**
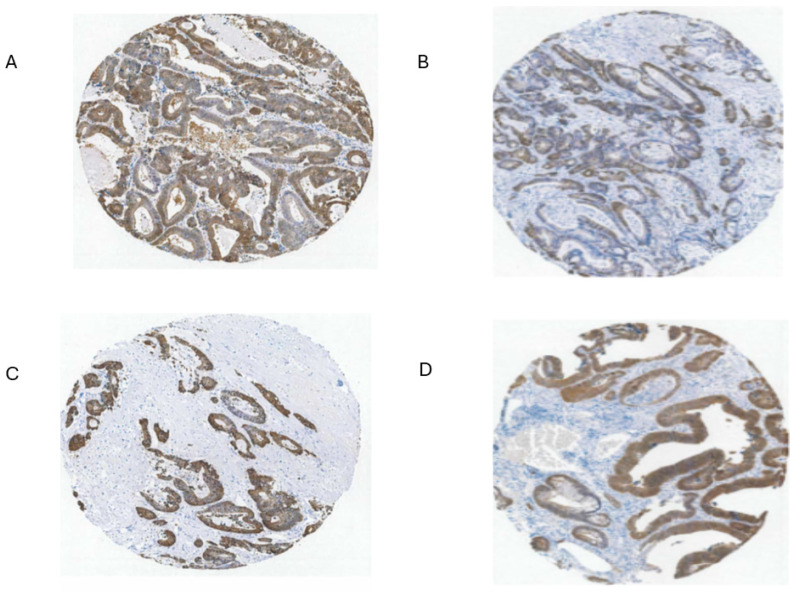
A set of GPX2 CRC IHC-stained samples showing some variation in staining intensity within the tumors (THPA, 2/5/2024). Panels (**A**–**D**) are IHC results from different patients.

Part of the issue in comparing normal to CRC using TPM/FPKM or standard IHC presentation (combined intensity and proportion scores), beyond lack of clear identification of the source cells, is the limited numbers of normal high-expressing cells and the evident increase in the proportion of expressing tumor cells in CRC samples (tumor purity) (Figure 3 and Figure 7) [29,30,44].

The upper range of the increase in tissue expression of 20-fold could be accounted for by the 4–5-fold increase in cell level expression and a 4–5-fold increase in the relative volume of the expressing cells in tumor samples, although many CRC samples seem to exceed this increase in proportion based on IHC at THPA (Figure 7). None of this might be relevant if the source tumor cells are not represented by the naturally high *GPX2*-expressing normal cells. The question of source cells is complicated by alterations in the GPX2 protein and mRNA expression pattern observed in mouse colon after dextran sodium sulfate-induced injury. Here, the zone of high expression was expanded to the luminal region, showing that many cell types in the colon can produce high GPX2 levels under stress [45]. There may be differences between mice and humans in the range of GPX2 protein expression in the ileum and colon. IHC of mice seems to show high levels of protein at the crypt/gland base and moderate levels of protein detected up to the villus tip/lumen, while one IHC study of human samples seems to show expression only in the gland base, and in a second study, the image is in gray tone, and it is unclear by inspection whether any protein was detected outside of the narrow range of high levels at the gland base [29,30,46]. The authors comment that GPX2 was detected in the ileum crypts and colon gland base and do not mention detection outside of those zones [30]. In a third case, GPX2 was detected at the lumen with decreased levels from the colon gland base while confined to Paneth cells in the ileum [31]. Enlargement of the image of human small intestine GPX2 IHC in Figure 3 hints at very low expression in patches in the upper crypt and up to the villus tip; however, the call by THPA is that cell types other than the Paneth cells are negative for expression.

### 3.5. Efforts to Assist Investigators in Selection of Cell Lines

**Figure 8:**
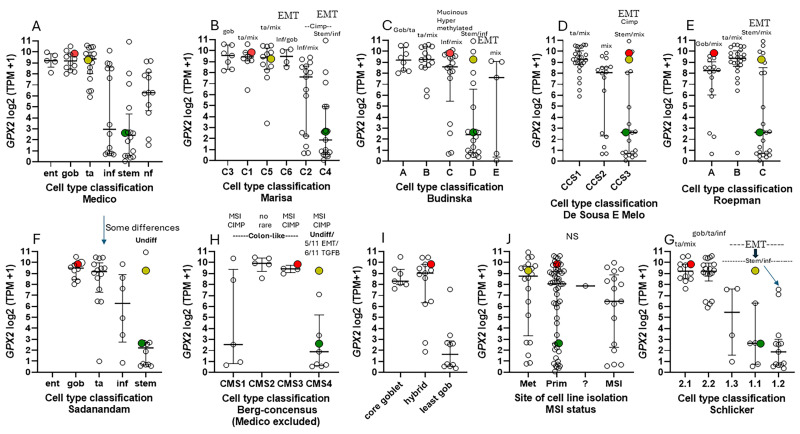
Classification of cell lines in various schemes into CRC subtypes with *GPX2* expression levels (log2 TPM+1) [6,7,8,9,10,11,12,13,14,15]. Medico et al. [6] have the largest set of lines, although only 65 are found in common with DepMap. NF in Panel (**A**) indicates cell lines in DepMap and not in Medico et al [6]. Medico et al examines cell lines, exclusively, and expands on the classification scheme of Sadnandam et al. [6,7], with some differences arising between the two with no real impact on the grouping of low and high expressing lines. The Medico/Sadnandam group names are carried over into the other schemes (Panels (**B**–**H**); ent-enterocyte-like; gob-goblet-like; ta-transit amplifying-like; inf-inflammatory-like; stem-stem cell-like; nf-not found in Medico et al.) since the information was obtained from processing by Medico et al., except for Panels (**F**,**H**,**G**). Properties of the groups are listed above in Panels (**B**–**H**) (EMT—epithelial to mesenchymal transition; Cimp-CpG island methylator phenotype; and MSI—microsatellite instability). (Panel (**H**)) is a consensus set of the groups in (Panels (**B**–**G**)) that contained 34 cell lines in total with the overlap with DepMap shown [13]. (Panel (**I**)) shows groupings obtained using markers differentiating adenocarcinoma and mucinous adenocarcinoma portrayed as a spectrum of goblet-like properties [14]. (Panel (**J**)) shows the origin of the cell lines between the primary tumor (Prim) and metastatic sites (Met). ? Not clear. MSI+ cell lines. NS: no significant differences in *GPX2* expression levels among groups. Oversized dots indicate cell lines HT29 (red), SW480 (dark green), and SW620 (yellow). The stout arrow in Panel (**G**) indicates that EMT is a strong property of the 1.1 class, and the thin arrow indicates that class 1.2 has the most inflammatory-like cell lines.

The suitable selection of cell lines for CRC studies is made possible by analysis of CRC for driver and passenger mutations (classical analysis) and later efforts to classify tumors by small- or large-scale analysis of mRNA expression profiles and IHC, often combined with mutation profiles and epigenetic modification profiles; Cimp, CpG island methylator phenotype; and MSI, microsatellite instability (CMS, CCS, CRIS, iCMS, and others) [7,8,9,10,11,12,13,14,15,47,48,49]. In some cases, cell lines were similarly classified in attempts to match them with CRC types (Figure 8) [6,7,8,9,10,11,12,13,14,15].

**Figure 9:**
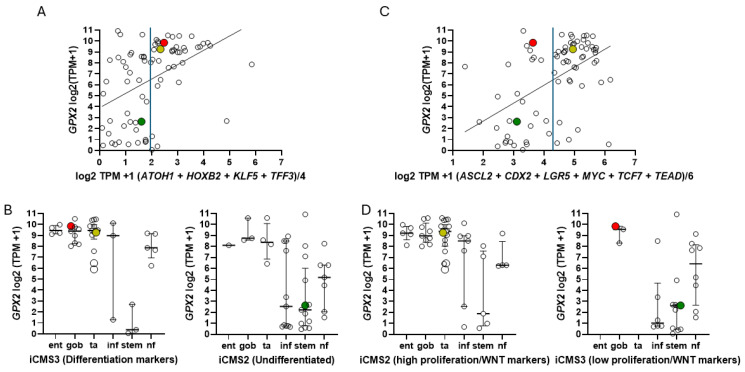
Panels (**A**,**B**) show the correlation of *GPX2* levels with the average of 4 differentiation markers (**A**) and a classification plot for cell lines with high or low expression of differentiation markers based in the iCMS system (**B**) [15]. In both panels, HT29 is indicated in red, SW620 in yellow, and SW480 in dark green. Panels (**C**,**D**) show the correlation of *GPX2* expression levels with the average of *ASCL2* + *CDX2* + *LGR5* + *MYC* + *TCF7* + *TEAD* expression in cell lines (**C**) and in a cell line classification plot with high or low proliferation/WNT marker cell lines (**D**). In Panels (**A**,**C**), the vertical blue lines denote the rather arbitrary division of low vs. high marker expression. While isogenic lines, SW480 and SW620, are separated in Panels (**B**,**D**), the isogenic pair, GP2D and GP5D (large white circles), remains associated.

The key point is that CRC tumors can be divided into up to six classes, and, in addition to differences in gene expression profiles, each category has a tendency for certain driver mutations, levels of copy number variation, CIMP profiles, MSS/MSI status, and, important for this discussion, differences in poor relapse free survival (RSF) and metastasis. The classification systems of the various groups, while showing some discrepancies, are similar enough that they were consolidated by Guinney et al. into a consensus classification system (CMS; Figure 8H; involving groups in Panels B–G) [49]. The availability of high throughput transcriptomics drove much of the classification, being relatable to cellular phenotypes and the clinical behavior of tumors. The consensus classification uses a neutral terminology (CMS1-4); however, this is sometimes phrased as enterocyte- (Ent-), goblet- (Gob-, CMS3), transit amplifying- (TA-, CMS2), inflammatory- (Inf-, CMS1), and stem-like (Stem-, CMS4), suggesting gene expression level affinities to normal cell types of the colon (Figure 8A,F) [6,7]. CMS1 tumors tend to be hypermutated and are largely MSI and CIMP+, with *BRAF* mutations and low copy number variation. CMS2 has high copy number variation, prevalent *APC* and *TP53* mutations, and is MSS. CMS3 has a high prevalence of *KRAS* and *APC* mutations with some *TP53* mutation, moderate levels of CIMP+, and is MSS. CMS4 has high copy number variation, is MSS, and has moderate levels of mutation in *KRAS*, *APC,* and *TP53*, and is largely CIMP-. Guinney et al. also provide an extensive analysis of differential properties for WNT/MYC target expression (CMS2), metabolic processes (CMS3, sugars, and fatty acids), immune processes (CMS1), and EMT/TGF-β pathway activation (CMS4), among others. Consistent among the individual classification schemes and preserved in CMS is the finding of poor relapse-free survival in CMS4 and, in Guinney et al., overall survival as well. CMS4 is also noted for metastasis, associated with high EMT pathway expression. CMS1 is associated with poor survival after relapse. These last factors will be important in the discussion of cell line choices, and it is worth double checking potential cell line choices for consistency with the MSS/MSI, Cimp+/−, copy number variation, and mutation status properties of the related tumor class. All of this was intended to produce better prognosis criteria, precision therapies, and inform the process of cell line selection for pre-clinical testing of such therapies, with Medico et al. concentrating on the choice of cell lines (Figure 8A) [6]. An anomaly in the CRC nomenclature system of Medico et al. (inherited from Sadanandam et al.) is that in applying a system based on markers for normal cell types to cancer, some classical stem cell and WNT markers are associated with the TA-like class (*ASCL2*, *RNF43*, *ZNFR3*, and *AXIN2*) (Figure 8A) [50]. Sadanandam et al. stated that the main characteristics of the Stem-like CRC class were high expression of myoepithelial and mesenchymal genes and lack of differentiation. While they also say this CRC class has high WNT marker expression, this was not found to be a consistent property of the cell lines that were sorted into this category based on the stripped-down iCMS marker set and was not supported by the findings of the CMS analysis for tumors of the CMS4 set (Figure 9) [15,49]. Lack of differentiation with high WNT marker status was oddly found in cell lines scattered across the Ent-, Gob-, Inf-, and TA-like classes.

One notable outcome of the collective efforts was the relatively consistent classification of some cell lines into epithelial to mesenchymal transition (EMT)/TGFB pathway activation/undifferentiated/low WNT expression class (Figure 8). A characteristic of this set of cell lines in most classification schemes is low *GPX2* and *NOX1* expression (median levels—4.35 TPM and 0.1 TPM vs. 588 TPM and 9.4 TPM in other classes, respectively). There is some inconsistency among the classification schemes for placement of cell lines, and low-expressing lines are sometimes split among 2 or 3 classes within schemes (Figure 8A–I). For CMS, only 34 cell lines have been independently classified, with 23 found in DepMap (Figure 8H). The results largely recapitulate Medico et al., so the results of the Medico et al cell line classifications will be used for further discussion. Since the main goal was to observe how high and low *GPX2*-expressing cell lines were sorted based on the various CRC typing methods, extensive statistical analyses were not performed. For the Medico et al. analysis, Ent-, Gob-, and TA-like *GPX2* expression levels are not different and Inf- and Stem-like are not different. Inf and Stem are significantly different from Ent-, Gob-, and TA-like (Figure 8A) (*p* ≤ 0.021; Mann–Whitney; log2 transformed data directly from DepMap; PRISM 9.3). For CMS sets, CMS1 is not different from any other set; this classification corresponds to the Inf-like cell lines of Medico et al. [13,49]. CMS2 (largely TA-like) and CMS3 (mix of Gob-, TA-, and Inf-like) are not different, while both are different from CMS4, corresponding to a mix of the Stem-like and Inf-like sets of the Medico et al. classification (Figure 8H) (*p* ≤ 0.0056). The mucinous adenocarcinoma marker analysis showed the least goblet set to be different from the core goblet and hybrid sets, which were not different (Figure 8I) (*p* < 0.0002). In the case of cell line origin, primary tumor, or metastases, and MSI, no significant differences were found (Figure 8J).

Possible evidence of deep secretory-like derived cell lines (colon Paneth cell equivalent; possible candidates for high *GPX2* expression) in the DepMap set can be found using the differential markers indicated in Sasaki et al. [33]. It is not clear whether such cells would have any tumorigenic capacity or whether any of the original differential markers of the postulated cell type would remain detectable when or if a transition like that of Paneth cells acquiring stem cell properties occurred. Presumably having acquired high WNT marker status while retaining some expression of differentiation markers, *CD24*, *REG4*, *MMP7*, *DLL4*, *EGF*, S*PDEF*, and *ATOH1*, they could be represented by 20 of 40 cell lines of the Medico et al. classification scheme that overlap with DepMap, excluding Stem-like and the NF categories, with *GPX2* levels > 190 TPM and 1 Stem-like cell line at 1 TPM. This is pure speculation, as mice were studied for evidence of deep secretory cells, and they are not yet documented in human samples.

### 3.6. The Issue of Low GPX2 Expressing CRC-Derived Cell Lines and Circumvention

**Figure 10:**
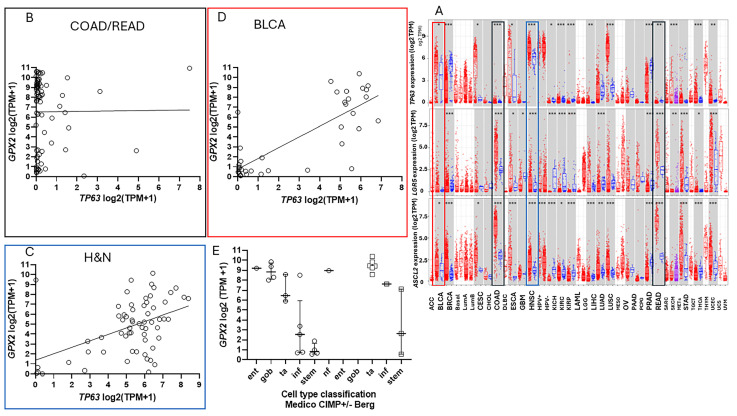
Normal and cancer tissues expression of *TP63* vs. *ASCL2* and *LGR5* by source. Black rectangles denote COAD and READ, red-BCLA and blue-HNSC cancer (Panel (**A**)). (Panels (**B**)) shows the lack of *TP63* expression in CRC-derived lines, comparable to results in (Panel (**A**)), while (Panels (**C**,**D**)) show generally higher expression of *TP63* in cell lines from H&N cancers and BCLA, with some indication of a positive correlation with *GPX2* expression. (**E**) Distribution of CIMP+ (circles) and CIMP- lines (squares) in Medico et al. classes with *GPX2* levels. *, **, *** in panel (**A**) denote *p*-values for the significance of the difference in levels between respective normal and tumor sets; *, 0.05; **, 0.01; ***, 0.001.

Where do low-expressing cell lines come from? In cancers of the GI tract, *GPX2* expression is confined to uniformly high levels, more so for COAD and READ (Figure 1A). In other cancers, the range of expression is enormous, possibly explaining the establishment of some cell lines with low levels. Some low-expressing cell lines from CRC have mutant APC, and others with wild-type APC have high expression of at least one WNT ligand (DepMap) (Figure 4). Thus, the WNT dependence of *GPX2* expression, demonstrable in some CRC-derived cell lines and mice, does not seem to be the reason for low expression [51]. Analysis for *BRAF*, *KRAS*, *PIK3CA*, and *TP53* mutations did not reveal any association with *GPX2* expression levels in CRC-derived cell lines (DepMap). *SMAD4* mutations are more frequent among the high *GPX2* expressing lines but also found in intermediate and low expressing lines (11 of 14 cell lines with mutations, *GPX2* TPM > 64; 22% of high expressing lines, 11% of low expressing lines; *p* = 0.36). *GPX2* expression levels are linked to NRF2 activation [2]. There are not potent activating agents in standard culture media, and this could be one factor in low *GPX2* expression. TP63 is one of the major drivers of *GPX2* expression in other tissues and cancers, and there is sometimes a demonstrable correlation between *TP63* and *GPX2* levels among cell lines derived from these other cancers (Figure 10) [52]. *TP63* expression is notably absent in all but a few CRC-derived cell lines being replaced by WNT (Figure 10) [51].

Because the classification of cell lines was performed after the fact, there will be discrepancies with the properties of the related tumor types. One of the more glaring issues is the excess of CIMP+ cell lines in the Stem-like (CMS4) class. There is some trend for CIMP+ cell lines of the Inf- and Stem-like categories to have low *GPX2* expression (<6 TPM; 4 of 5 Inf-, and 4 Stem-like; note, CIMP+ is not a general property of the corresponding CMS4 class for Stem-like tumors and is a property of the corresponding CMS1 class for Inf-like tumors), while CIMP+ lines in the Ent-, Gob-, and TA-like sets retain higher expression (≥60 TPM; Figure 10E). This might indicate suppression of *GPX2* gene expression by this repressive mark [53,54]. Two CIMP-, Stem-like lines also have low *GPX2* expression, so it is not clear what the relations are: CIMP+ in the Inf- and Stem-like categories specifically leading to low *GPX2* expression, or more likely the condition of being Inf- or Stem-like leading to low *GPX2* expression as the primary effect, while the CIMP+ condition is an incidental feature overrepresented in CRC cell lines relative to tumors [13,53,54]. One criticism of cell lines is there is a tendency for excess CpG methylation (a little less extreme in CRC-derived lines, 5-fold), although methylation sites found in CRC tend to be preserved. This was attributed to myriad effects of cell line derivation and cell culture conditions, to which loss of variability of CRC-derived lines compared to CRC was also blamed [53,54,55].

**Figure 11:**
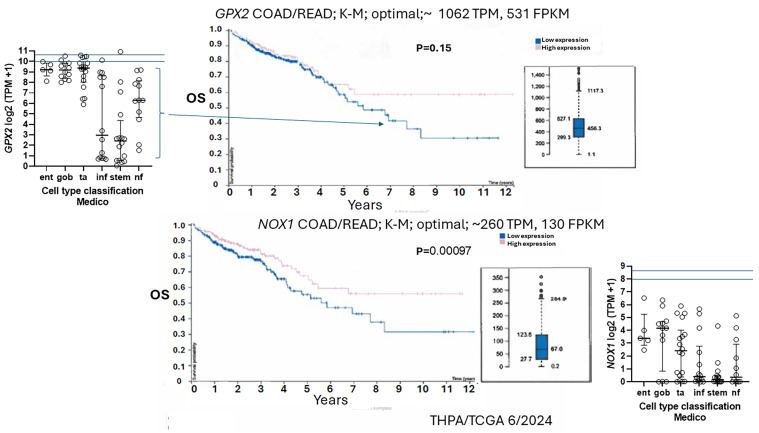
Kaplan–Meier plots from THPA (6/24) assessing OS prognosis based on *GPX2* and *NOX1* expression levels. The approximate cutoff levels are indicated as horizontal lines on the cell line classification/expression levels plots. The upper line in the *GPX2* and *NOX1* plots is a correction to the TPM for the median tumor purity for CRC samples, made by multiplying the TPM (derived from TPM ≈ FPKM × 2) by the empirically determined conversion factor from ref. [1] by 1.6, correcting to a tumor purity of 100% based on median tumor purity for CRC samples of 0.62 for comparison of tumor samples to cell line TPM values [1,44]. The bracketed cell lines in the *GPX2* plot are those with expression levels below the cutoff, although cell lines with values less than log2 8 are not really indicated by tumor data. The inserts are the distribution of tumor sample expression levels in FPKM.

Based on the consistent EMT expression profile, it might be conjectured that the low *GPX2*-expressing cell lines derive largely from metastases. This is not the case (Figure 8J). In fact, one study attempting to resolve the roles of GPX2 in tumorigenesis and metastasis showed that while HT29 cells with *GPX2* knocked down showed poor growth as xenografts in mice, they could metastasize, although demonstrating a lower potential (*GPX2* levels in HT29 at 1010 TPM—averaging DepMap and THPA values; about 20-fold knockdown; *NOX1* 16 TPM) [56]. However, follow-up analysis showed that the metastatic tumors were a subset that either did not fully respond to the knockdown or re-established higher *GPX2* expression in the process (IHC). Since HT29 cells were classified as CCS3 (Figure 8D, EMT set; poor relapse-free survival (RFS)), the authors went on to examine RFS in CCS3 cancers stratified by low and high *GPX2* expression, with high expression being unfavorable; questionable significance (TCGA/THPA does not confirm any prognostic relationship between *GPX2* and overall survival (OS), lumping all classes together; Figure 11).

They noted that the median level of *GPX2* expression in CCS3 CRC subsets was ~512 TPM (correcting for median tumor purity in CRC-820 TPM) much higher than the 5 TPM characteristic of most CCS3-type cell lines (CCS1 vs. CCS2 and CCS3, *p* ≤ 0.0006; Figure 8D) and that the variation in expression levels was only 4–5-fold among the CCS3 tumors [5]. The relevance of a 20-fold knockdown is questionable in the context of probing RFS. It might be reasonable as a therapeutic goal given the general negative impact on tumor growth and metastasis. HT29 is generally not classified into the EMT/TGFB pathway activation/undifferentiated/low WNT expression set among classification schemes (Figure 8). The cell line is somewhat differentiated (some *MUC2*, *TFF3* expression, and high *LYZ*; *ATOH1*, *HOXB2*, *KLF5*, and *TFF3*; and iCMS), has somewhat low TGFB pathway marker expression (*CCN1*, *ITGAV*, and *TGFB*) and low EMT marker levels (*FN1*, *CDH2*, and *VIM*), while sharing low WNT marker expression (*ASCL2*, *CDX2*, *LGR5*, *MYC*, *TCF7*, and *TEAD*; iCMS) characteristic of the EMT sets (Figure 9) [57,58,59]. HT29 can undergo differentiation in culture with the substitution of galactose for glucose in the media, with some cells exhibiting goblet cell-like morphology and mucin production [60]. Slightly lower GPX activity in HT29 was associated with differentiation by 5-Fluorouracil [61]. This study predates wide-spread recognition of GPX isoenzymes, so GPX1 and GPX2 were not separately examined. As a side note, galactose increases mitochondrial H_2_O_2_ release, hinting at a role for ROS in the differentiation process along with the reported lowering of GPX activity [62].

Nine cell lines with higher *GPX2* levels are classified by two or more of the schemes referenced in Figure 8 into the EMT/undifferentiated categories: CACO2 (3 schemes), CL40 (2 schemes), LOVO (3 schemes), LS180 (2 schemes), SKCO1 (2 schemes), SNU1040 (3 schemes), SW620 (5 schemes), SNU503 (5 schemes), and SNU1033 (3 schemes) with *GPX2* expression levels of 118, 918, 290, 296, 1711, 1020, 648, 2040, and 318 TPM, respectively (average, DepMap, and THPA) (Table 1) [7,8,9,10,11,12].

There are discrepancies with and between Medico et al./Sadanandam et al. [6,7]; LS180 and SKCO1 are classified as Gob- by both, while SNU1040 is split Ent-/Gob- and SW620 is split TA-/Stem-like. This appraisal might be further limited by the suggestion that MSS tumors of the CMS4 class are most likely to metastasize, leaving CACO2, CL40, SKCO1 (metastasis), SNU503 (metastasis), SNU1033, and SW620 (metastasis) as representative cell lines with potentially high metastatic potential [15]. However, LOVO (MSI; metastasis) and HCT116 (MSI; low *GPX2* expression; identified in the EMT/undifferentiated classes in all schemes) were found to have metastatic potential in a mouse xenograft model rivaling that of SW620, a cell line derived from a metastasis with metastatic potential demonstrable in 2 mouse model studies [63,64]. There may be a few seemingly EMT/TGFB pathway activation/undifferentiated/low WNT expression category cell lines with *GPX2* expression levels approaching tumor levels, which seems desirable in testing the role of GPX2 in this class of tumors based on the commonly used suppression of *GPX2* expression in cell lines as a mode of study; notably, CACO2, SNU503, SNU1033, and SW620 for Stem-like cell lines. CL40 and LOVO might be as appropriate as high-expressing lines for the Inf-like class (Medico et al./Sadanandam et al.) [6,7]. Within these two classifications, paired high and low expressing lines may represent cases where the assumption that a major difference is in *GPX2* levels is somewhat justified and the use of forced over-expression and silencing might yield relevant findings, with the caveat that very low expression in cell lines is not yet supported by CRC expression levels and differences in expression levels of *CST6*, *EGFR*, *MYC*, *TP53*, *VIM,* and other putative redox-sensitive proteins may be present.

### 3.7. How Different Are CRC-Derived Cell Lines Sorted into Classes by Medico et al. for Expression Levels of Genes Encoding Proteins with Reactive Sulfhydryl Groups?

While the CRC-derived cell line sorting process is largely based on differential gene expression profiles, just how different are the cell lines for mRNA levels of proteins with known or suspected reactive sulfhydryl groups that impact function when oxidized? While the list of potentially affected proteins is large and growing, a few key proteins were selected for evaluation in DepMAP [2,65]. Uniformity and variability in expression levels were found across the groups of cell lines in the Medico et al. classification scheme (DepMap) [66,67,68]. Moreover, 14-3-3-gamma (*YWHAG*), c-ABL (*ABL1*), *ASK1*, *AKT1*, *AKT2*, *HIF1A*, *JNK2*, *JUN2*, *ERK2*, *KEAP1*, MAPKs, *NFKB*, Nucleoredoxin (*NXN*), p38 (*MAPK14*), protein kinase A (*PRKACA*), *PTEN*, *PTPN1B*, *SHP1*, *SHP2*, *SRC*, and *STAT3* have uniform expression levels across classes. Cystatin B (*CST6*), *EGFR*, *MYC*, *TP53*, and vimentin (*VIM*) show skewing for certain classifications; *EGFR* levels tend to be slightly higher (2–4-fold) in low *GPX2* expressing lines, enriched in Stem- and Inf-like cell lines, some with high *GPX2* levels, although a few very low expressing lines have nearly zero levels; 4-fold lower expression of *TP53* is associated with the high *GPX2* expressing TA-like cell lines compared to the other classes; high levels of *CST6*, 16-fold or more, are generally associated with the low *GPX2*-expressing Stem- and Inf-like classes; *VIM* is slightly skewed in favor of low *GPX2* expressing cell lines (2–4-fold), with 5 Stem-like low expressing lines and SW620, a high expressing Stem-like line (most schemes; TA-like in Medico et al. [7]; Figure 8 and Table 1), showing roughly 8-32-fold higher expression than the bulk. *MYC* shows slight skewing (2-fold) in favor of low *GPX2*-expressing Stem- and Inf-like cell lines. It could be argued that for most redox-sensitive proteins upstream of major pathways, there would be a uniform impact of ROOH modulation across CRC classifications with a few exceptions and that, in many cases, mRNA levels do not reflect the functionality of the proteins. However, this exercise can only be efficiently managed using the available mRNA expression data. *CST6*, *EGFR,* and *VIM* might be exceptions, where mRNA levels reflect function. The other point is that the downstream protein profiles may be variable among the CRC-derived cell lines based on the classification method; Medico et al. used 783 differentially expressed genes in their profiling [6]. That is, *GPX2* expression level differences generally occur in a context of broad gene-level expression differences and not in isolation. Forced overexpression and silencing of *GPX2* in one or a few cell lines may not reflect this situation; biasing results in favor of the impact of the consensus redox-sensitive proteins and downstream pathways, assuming these remain largely unaffected by the manipulations. However, it was shown above that there may be a few suitable pairings of high and low *GPX2* expressing lines for the Stem- and Inf-like classes. While the argument has been made that GPX1, PRDXs, and CAT impact should also be accounted for, a broader systems approach may be required to understand how *GPX2* expression alterations (and the other antioxidants) fit into the scheme of CRC and what drives the large-scale gene expression changes that include *GPX2*.

### 3.8. Intratumor Variation, Plasticity of Cell Components, Stromal Cell Interactions, and GPX2

There is a general trend for high *GPX2* expression in normal colorectal samples at the tissue level (Figure 2; ?). This appears to be achieved with a limited zone of high expression at the base of the glands [29,30,31]. There is disagreement on whether a low level of GPX2 expression extends to the top of the gland. While one study mentioned above showed individual CRC tumor cells to have elevated GPX2 protein levels relative to expressing normal cells in a somewhat small set, it is not clear that this is universal in CRC samples [29]. THPA GPX2 IHC sets show a few tumors where GPX2 intensity is quite variable in the epithelium, with portions of tumors showing almost no staining adjacent to strongly strained sections, and other lesser variation is also observed (Figure 7A,B). The unstained or lightly stained portions are likely the normal epithelial cell component of tumors (not considered as a source of CRC-derived cell lines) that occurs at an average of 24% of epithelial cells isolated from tumors in the iCMS analysis, presumably having low levels of GPX2 relative to the tumor cells [15]. The sample shown in Figure 7B might represent a tumor with a normal component above this average, while panels C and D seem to be below the average. The reverse staining pattern is unlikely given the low numbers of normal high *GPX2-*expressing cells, unless alterations in the tumor stromal cellular environment promote aberrant growth of normal cells along the lines of Paneth cells in tissue injury (postulated deep secretory cells in the colon) [30,32,33,34,35]. Whether any of this variation relates to the tumor cell component has not been systematically studied, so we would have no idea about how prevalent it is as a pattern and whether it relates to CRC subtypes [15]. If this involves the tumor component, a conjecture is that the CMS4/CCS3 class may be more represented, explaining the propensity for low *GPX2*-expressing cell lines from this class (Figure 8). This can be readily tested, and if the variation involves the tumor cell component, this could justify the use of low-expressing cell lines in studies.

*GPX2* expression in primary tumors may be dependent on factors secreted by CAFs and other stromal cells, and portions of the tumors may lack sufficient contact with these cells to support *GPX2* expression. Growth in primary sites seems to be somewhat dependent on stromal support [69,70,71]. Tumor cells seem to be plastic, altering their properties, which might include cellular factors affecting *GPX2* expression, separate from stromal factors [38]. Stromal dependency, while possible, is countered by the more general tendency of cell lines to retain *GPX2* expression in standard culture conditions and the idea that metastases can be established in the absence of stromal support (DepMap) [38]. It is estimated that only 10–15% of CRCs can supply cells that will establish cultures [72]. A general requirement for stromal support in primary tumors and re-establishment of this after metastases have seeded may explain part of this failure. In standard isolation of CRC-derived and other cell lines, fibroblasts are the main contaminant, and it takes several passages to eliminate them, or they may be employed as feeders until overgrowth is evident as a sign of independence of the CRC epithelial cells [73]. It is possible that a transition from stromal dependency to independent growth occurs by adaptation or selection as the fibroblasts are eliminated [73]. There are no broadly consistent patterns of gene expression among cell lines, as evidenced by the stratification into as many as six subtypes based on differences in expression, driver mutations, and epigenetic modulation patterns (Figure 8). It is possible to find many different combinations of driver and passenger mutations in the list of 77 CRC cell lines in DepMap [47]. Thus, the basis for culturing success is elusive and clearly has nothing to do with *GPX2* expression either directly or in the context of cell properties favoring *GPX2* expression.

A side project on *GPX2* expression could include adding CRC stromal factors back to cultures, or better, using stromal cells as feeders of the low-expressing lines looking for induction of *GPX2* levels to the range of 100–2000 TPM [73]. There is evidence that high *TGFB* expression in tumor cells, somewhat greater in low *GPX2*-expressing cell lines (DepMap), exerts a paracrine effect on the surrounding stromal cells, which in turn enhances metastasis of fibrotic-type CRC tumors [74]. This would have to be linked to a broader examination of gene expression alterations to avoid overinterpretation for any impact of GPX2 on subsequent cell line properties. Another point raised by the substitution of galactose for glucose in the culture of HT29 and the change in properties is the presence of additional carbon sources for intestinal cells, short-chain fatty acids, and glutamate [75,76]. The addition of short-chained fatty acids to culture media seems to evoke changes in cell line properties with a generally negative impact on growth [77]. Retinoic acid was found to increase *GPX2* expression in cell lines, particularly MCF7, a line that barely expresses *GPX2* under basal culture conditions [2].

As a general statement, based on Figure 2A and the IHC in Figure 7 (several other similar examples can be found at THPA), there is an expectation that CRC tumors will have high *GPX2* expression both at the tissue level and throughout the epithelium. A study attempting to understand changes in epithelial properties as CRC tumors develop from polyps found that Stem-like cells showed a consistent increase in *GPX2* levels by 3–5-fold as the malignancy progressed, relative to normal Stem-like cells, with elevation evident in the polyps [32]. This change in expression levels is coincident with the 3-5-fold increase in GPX2 protein level expression found by Brzozowa-Zasada et al. in the comparison of high-expressing normal cells and tumors [29]. *ASCL2* and *OLFM4*, stem cell markers, show increases in levels in the same cell population, while *HNF4A* has a biphasic pattern, decreasing from normal to the polyp phase then increasing dramatically in the CRC stage. *HNF4A* shows a slightly stronger correlation (R-squared, 0.48) with *GPX2* levels in cell lines than *ASCL2* (R-squared, 0.31) and *OLFM4* (R-squared, 0.18), but for all the residual deviation from the regression line is huge for a large fraction of lines. *GPX2* has been occasionally proposed as a stem cell marker, so that correlations to other such markers in CRC samples (*AXIN2*, *RNF43*, *ZNRF3*, and *SOX9*; no correlation with cell line *GPX2*, although all are upregulated in CRC) have been made before and associations with WNT proposed and established [32,50,51]. For *HNF4A*, the pattern is explained as yielding to WNT dependency early on, being an inhibitor of WNT, and fueling CRC later [32,78]. The number of independent samples in this study was very small, only three CRC samples, and unable to cover the spectrum of CRC classes. However, there is no indication that *GPX2* levels should be low in tumor samples.

Pathways that drive high *GPX2* expression, indicated by results from tumors, could be replicated in high-expressing cell lines with the provision that there may be some dependence on stromal factors in tumor expression that would not be carried over to cell culture. There are many pathways that impact *GPX2* levels, so finding a strong correlation with any single gene or pathway is unlikely [2]. Esworthy, Doroshow, and Chu presented an overview of genes and pathways that influence *GPX2* levels, and the count was at least 14 at that time [2]. NFR2, WNT, TP63, and RARE have been mentioned above. Others, including CD13, *ETS1/2*, *FOXO1*, *FOXM1*, *OXR1*, PI3K/*AKT,* and *STAT3,* do not show a strong correlation with *GPX2* expression in cell lines, and *NKX3-1* is not expressed in the colon. *FOXM1* is upregulated in CRC, and *FOXO1*, *EST1,* and, particularly, CD13 (*ANPEP*), are downregulated (TIMER2.0). HIPPO/YAP is unique, reported to strongly suppress *GPX2* expression. Like *HNF4A*, which is in a cooperative feed-back loop of expression with *HNFA1* and therefore involved with the NOTCH pathway, a few master regulators of CRC pathways, *MYB*, *REG4*, and HIPPO/YAP, show a reasonably strong correlation with *GPX2* expression, markers of YAP activation, and related genes, a negative effect [78,79]. *MYB*, which is expressed in other tissues, shows marginal upregulation in CRC with a fairly strong positive correlation with *GPX2* in cell lines (R-squared, 0.498) [80]. *REG4* is expressed in normal GI-tract tissues and is not uniformly upregulated in CRC [33]. The correlation with *GPX2* in cell lines is weaker (R-squared, 0.22). *YAP1* is widely expressed at uniform levels and is not upregulated in CRC. Expression levels of *YAP1* and *TAZ* (co-activator) are also uniform across cell line classes. Markers of YAP/TAZ pathway activation show various degrees of negative correlation with *GPX2* expression in cell lines, *CCN1* (CYR61), with an R-squared of 0.64 (DepMap). *CCN1* is also a marker of the TGFβ pathway, providing two possible reasons for the strong correlation [17]. YAP activation might occur in cell culture, as it senses mechanical cues related to cell shape and cell spreading [13,81]. Thus, low *GPX2* expression in the Stem-like category of cell lines could reflect an impact of conventional cell culture. YAP activation was tied to downregulation of *GPX2* in LUSC through TP63, which is not expressed in colon or CRC-derived lines, requiring another mechanism of action (Figure 10) [82,83].

### 3.9. NOX1 in CRC and Links to GPX2

Expression of *NOX1* among CRC-derived cell lines shows a similar pattern of variable expression, with a greater proportion of lines exhibiting nearly zero expression relative to *GPX2* (Figure 5). Very high *NOX1* levels are a unique property of normal colon/rectum and CRC, with a narrow range of variability in expression levels in normal and a very broad range of expression in CRC, so that low expression in cell lines is not discrepant as with *GPX2* (Figure 2 and Figure 5). *NOXO1*, but not *NOXA1*, shares unique high expression in the colon/rectum (TIMER2.0). The only linkage in co-expression might be that high *NOX1* levels are associated with high *GPX2* levels as observed in cell lines (DepMap) (Figure 5). *NOX1* mRNA and NOX1 protein expression occurs at the crypt/gland base like GPX2 [26,27,28]. Whether co-expression occurs in the same normal cells is not clear. The absence of NOX1 (*NOX1*-KO) in mouse colon has some impact on the distribution of differentiated cells (goblet cells) and proliferating cells, favoring differentiation [84]. This suggests that *NOX1* might be expressed in the TA compartment, which is the cell line assignment of Medico et al. [6]. In the absence of GPX1 and GPX2, NOX1 activity produces pathology in the form of excess crypt/gland apoptosis, which may be dependent on the presence of microbiota and the composition, providing one rationale for very high *GPX2* in the colon/rectum [24,25,85,86]. The pattern in CRC-derived cell lines shows that co-expression of *GPX2* and *NOX1* is likely in cancer as a property of several, but not all, high WNT-expressing and differentiated cell lines (Figure 5 and Figure 11). Unlike *GPX2*, *NOX1* expression levels are considered prognostic by TCGA/THPA for OS as of 1/6/2024 (Figure 11). *NOX1* stratification yields a significant effect on OS, with high expression being favorable. The cut-off for the prognosis call is ~262 TPM (420 TPM, correcting for median purity values), which is greater than two times more than even the highest expressing cell line (Figure 11). There is the question of whether NOX1 would be active in any cell lines. One paper tried to address this by showing that a handful of CRC-derived cell lines at the top of the expression range might be capable of significant spontaneous and PMA-induced production of superoxide from NOX1 [87]. The knockdown of antioxidant enzyme expression in CRC cell lines might reveal activity as increased levels of apoptosis that should relate to *NOX1* levels, if true.

### 3.10. Low GPX2 Expression in Cell Lines, Redux

Returning to the issue of how to select cell lines for pre-clinical studies of the involvement of GPX2 in CRC, a major question is whether to use low-expressing lines for studies. The observation of low GPX2 protein levels in portions of tumor samples may be insignificant as a consideration in CRC studies, representing normal cells, and low-expressing cell lines may be of little interest and utility in CRC studies despite their otherwise interesting properties. Plasticity is noted to occur in cancer stem cells. Single-cell analysis found a low incidence of multiple subtype signatures in individual tumors, probably based on one sample per tumor [15]. Another study showed that multiple sampling within metastatic tumors revealed unrecognized heterogeneity [88]. Plasticity in CRC tumor stem cells, the finding of a few tumors with multiple signatures, and the likelihood of regional variation within tumors suggest that the coexistence of low- and high-expressing cells in tumors could occur with selection for alternate states a possibility [38]. *GPX2* expression might not be an essential feature of tumor progress, only a legacy of expression potential. This might thwart efforts to use modulation of *GPX2* levels to achieve therapeutic ends. There is possibly only one example of such behavior in established cell lines, and there is a trend for consistent increases in *GPX2* expression along what is termed the malignancy continuum, suggesting a singular upward arc during tumorigenesis for Stem- and TA-like cell types [32]. Cell lines SW480 and SW620 are isogenic; the former were isolated from the primary tumor and the latter from a lymph node metastasis [89]. Among the many differences, the *GPX2* level in SW480 is ~5.7 TPM and in SW620 is ~648 TPM (averaging results from DepMap and THPA); SW620 has much higher expression of *LGR5* and *ASCL2* and slightly greater signs of differentiation, while they share very low *NOX1* expression (Figure 5 and Figure 9). In two studies of metastatic potential in mice, SW620 demonstrated a much greater ability than SW480 [63,64]. Comparing *GPX2* levels (DepMap) to the reported metastatic ability among six cell lines used in one of the two studies did not show any trend, suggesting other properties of those lines and possibly SW480 and SW620 were responsible [64]. The paired lines, GP2D and GP5D, retain similar, somewhat low *GPX2* levels, 87 TPM (83 TPM averaging DepMap and THPA) and 59 TPM, similar proliferation and differentiation marker status, and low *NOX1* levels (Figure 5 and Figure 9B,D). Both were derived from the same primary tumor and exhibit some different properties in culture, including responses to EGF ligands and spontaneous vs. induced EMT [90]. Supplementation of EGF ligands to culture media could be one more item to explore for impact on *GPX2* expression in addition to short-chained fatty acids and retinoic acid. Other pairings exist or may exist; however, one member of the pair does not appear in the DepMap or THPA databases, or the suggested pairing is based on later supposition and not the original description (DLD1, HCT15; primary; similar low *GPX2* and *NOX1* expression; multiple sources support pairing) (Figure 5) [6]. CRC organoids might provide a means to explore the plasticity issue [56].

## 4. Summary

In general, it appears that cell line selection for studies should account for CRC subtypes, selecting across broad gene expression profiles, different driver mutations, and epigenetic modulation. There is sufficient information and enough cell lines typed to account for most of the variation across CRC classes (DepMap and THPA) [6,7,8,9,10,11,12,13,14,15,47]. Medico et al. sorted 150 cell lines, and investigators could independently measure relative *GPX2* expression in the lines not covered by DepMap to include other combinations. Note that RT-PCR results from individual studies are often at variance with DepMap/THPA [1]. An investigation into this suggested that DepMap/THPA values were generally reliable guides for predicting GPX protein and activity levels in cell lines at a gross level, while the differences observed between DepMap and THPA reveal uncertainty for fine distinctions (Figure 4C,E) [1]. It is recommended to use at least two cell lines, one low and the other high expressing, from the DepMap collection to provide reference values when looking at other cell lines [1]. A study might need to use four or more lines to sufficiently account for the major variation among CRC classes, depending on the goals.

Classification systems tend to place high *GPX2* expressing and low *GPX2* expressing cell lines into different categories, and this may restrict the implementation of the common research scheme of overexpression in low expressing lines and knockdown of high expressing lines (Table 1), although some methods place both high and low expressing lines across classifications (Figure 8). As to using cell lines to model cancer etiology, the top-down model would suggest a likelihood that the source cells lacked *GPX2* expression and gained it as a function of progress along the malignancy continuum [32,39]. The bottom-up model does not guarantee that the source cells had high expression; however, this is a possibility, and cells in this area might be likely to acquire *GPX2* expression in an early hyperplasia phase based on evidence that NOX1 upregulation and activation are involved in the shift of gland base stem cells from quiescence to active cycling [91,92]. As of this time, there is no clear association between the few high-expressing cells found at the gland base and CRC. Until the plasticity and stromal contribution issues are resolved, cell lines with very low *GPX2* expression are something of an enigma, particularly with the trend for fully developed CRC to have higher *GPX2* expression than its antecedents [32]. Cells shed from primary tumors may lack *LGR5* expression and regain it after they seed metastases [38]. Likewise, it seems possible that this pattern might occur for *GPX2* based on the study by Emmink et al. [56]. The example of SW480 and SW620 suggests that primary tumors could have low-expressing cells and still produce high-expressing metastases [63,64,83]. This might reflect heterogeneity in the primary, plasticity, and the malignancy continuum arc as it relates to metastasis or just be an artifact of cell culture [55]. There is no uniform difference in *GPX2* expression patterns for cell lines isolated from primary and metastatic sites (Figure 8J). In lung adenocarcinoma, lower RFS seems to be related to persister cells that maintain higher levels of *GPX2* expression, which may be essential for the resilience of this cell type both in the quiescent and cycling states [93,94]. This is consistent with the findings that higher *GPX2* expression is associated with worse RFS in CCS3 CRC and inconsistent with the apparent isolation of low-expressing cell lines from CCS3 tumors (Figure 8) [56].

Based on the model for GPX1/2 action relative to the postulated PRDX relay (Figure 1), the main factors in CRC are ROOH signaling, in which NOX1 may have a major role, the GPX:PRDX ratio that would modulate the ROOH signaling potential, and the protein expression pattern of the cell type that would dictate the response to the ROOH signal. Another consideration is that PRDX reduction may be hampered by slow interaction with relay targets and a possible bottleneck with thioredoxin/thioredoxin reductase resulting in temporary inactivation [4,95,96,97]. GPXs may fill in for the loss of antioxidant capacity while PRDXs regenerate.

The relative TPM levels among *GPXs* and *PRDXs* are intended only as rough guides to comparative function, a starting point for thinking about where GPX2 might have a significant role and for choosing cell lines. PRDX1 and PRDX2 are estimated to have initial ROOH rate constants 2–5-fold over that of GPXs, while PRDX5 is estimated to be 10–100-fold less efficient [3,4]. This is coupled with the generally greater abundance of PRDXs [1]. The TPM counts can be modified based on these considerations to more realistically model relative antioxidant potency. On the other hand, the estimates of relative normal colon/rectum and COAD/READ tissue levels underrepresent *GPX2* (20–27% total TPM) by including expression of *PRDX1-6* and *GPX1* outside of the epithelial compartments; *GPX1* and *PRDX1-6* follow the pattern of β-actin in Figure 6. Attempting to correct for this revises the estimates for *GPX2* to 35–50% of total TPM; downplaying the contribution of PRDX5 by 10-fold and allowing the higher rate constants for PRDX1 and PRDX2, suggests GPX2 could be nearly at parity with PRDXs for ROOH consumption. In much of the normal epithelium, high GPX2 expression is not found, and in portions of some tumors, this is seen as well, yielding additional upward revision (Figure 1B, Figure 3, and Figure 7) [1,29,30]. *GPX2* levels equal to or greater than 35% of total antioxidant gene TPM are found in 10% of CRC-derived cell lines (up to 45%) (Figure 4). Applying the rate constant corrections to 5 of the top-expressing cell lines suggests that GPX2 could account for 20–27% of total ROOH reduction, up to 30%, including GPX1. The fraction of ROOHs going through GPX1/2 is significant because it could divert ROOHs from signaling via the PRDX relay (Figure 1A). Alteration of GPX2 levels is significant if it impacts the fraction of the total ROOH reduction capacity; that is, no commensurate changes occur among PRDXs. This is why information on GPX1/2 and PRDX1-6 is required in these studies to assess the actual impact. In the case of NOX1, if the predominant signaling is based on superoxide and H_2_O_2_ is only a by-product, having high levels of GPX1/2 could prevent unintended signaling (Figure 1B) [98].

Factoring in *NOX1*, possibly no CRC-derived cell lines would qualify for selection in studies (Figure 11). These considerations add more levels of uncertainty in evaluating the possible role of GPX2 in cancer and general antioxidant function, particularly using cell lines, which by and large fail to replicate the high *GPX2* and *NOX1* levels of COAD/READ. CRC and ESCA/STAD are among the better cases for finding representative cell lines [1]. LIHC-derived cell lines show vastly lower *GPX2* levels than the tumors (Figure 2).

## 5. Conclusions

Finally, no definitive answer can be provided on how to study *GPX2* in CRC. One question is whether forced overexpression or silencing of *GPX2* is the same as alterations to cells that result in changes to *GPX2* levels, where there might be changes to the potential target proteins as well. The study by Emmink, et al. suggests that suppressing *GPX2* in cancers with high expression (>820 TPM; based on HT29; the levels in the CRC-derived organoids used were not provided with the default from databases being high expression) might suppress growth and metastatic potential [56]. The study involved both the HT29 cell line and organoids cultures from 2 CRC subjects, giving it some veracity. They did not provide any information on CRC subtypes of the source for the organoids, and the in vitro culture conditions of the organoids were not typical, lacking WNT activators, Noggin, RSPO, etc. [99]. There are several other *GPX2* and CRC papers that collectively suggest that *GPX2* levels should impact CRC progress. More generally, in experimental studies, high expression seems to favor tumor progress, with a range of proposed pathways being impacted [1]. There are some conflicting findings as follows: high expression repressing the prostaglandin and lipoxygenase pathways and subsequent growth in vitro, while the same condition supported tumor growth in mice and suppressed mutation, which might disfavor immune checkpoint therapies; low expression suppressed azoxymethane-induced CRC in mice [100,101]. Prognosis analyses conflict with TCGA/THPA suggesting no impact on OS collectively, while focusing on the EMT subtype suggests high *GPX2* expression in this subtype may yield poor prognosis for RFS (Figure 11) [7,8,9,10,11,12,13,14,15,56]. CRC is complex, and the issues of plasticity and stromal involvement further complicate the situation, suggesting that multiple pathways could be impacted by GPX2 action and contribute to mixed research results. If it is worth the effort, research must account for the complexity and current uncertainties surrounding CRC, and this would involve careful selection criteria for cell lines that would go beyond *GPX2* levels and employ the use of multiple lines in any single study. Alternatively, reading this report might convince many that using cell lines is not a good approach to understanding CRC or the role of GPX2.

## Figures and Tables

**Table 1:** EMT/undifferentiated cell lines with high GPX2 expression.

Sadanandam	Marisa	Budinska	de Sausa	Roepman	Schlicker	MSS	MET	APC mut
SNU503	T84	SNU1040	SNU1040	SNU1040	CACO2	SNU1033	SNU503	SNU1033
SW620	SNU407	SNO503	HT29	CL40	LOVO	SNU503	LOVO	SNU503
	LOVO	CACO2	SNU81	SKCO1	SW1417	CACO2	SW620	LOVO
	LS180	LOVO	SNU1033	SNU1033	SW620	SW620	SKCO1	SW620
	HT115	C84	SNU503	SNU503		CL40		SKCO1
	LS411N	SW620	SW620	LS180		SKCO1		CL40
	SNUC41			SW620				SNU1040
	SNU1033			COLO201				
	SNU503							
	CACO2							
	CL40							
	SNU61							
	SNU1460							
	SKCO1							
	SNUC4							
	CL34							

Color coding indicates cell lines appearing in multiple classifications; see Figure 8 and main text. MSS-microsatellite stable; MET-cell line derived from metastasis.

## Data Availability

All data can be found in the referenced public databases and cited papers; inquiries can be made to R.S.E. (email: sesworthy@coh.org).
